# Cigarette smoke induces endoplasmic reticulum stress and the unfolded protein response in normal and malignant human lung cells

**DOI:** 10.1186/1471-2407-8-229

**Published:** 2008-08-11

**Authors:** Ellen Jorgensen, Andy Stinson, Lin Shan, Jin Yang, Diana Gietl, Anthony P Albino

**Affiliations:** 1 Public Health Division, Vector Research LLC, New York, NY, USA

## Abstract

**Background:**

Although lung cancer is among the few malignancies for which we know the primary etiological agent (i.e., cigarette smoke), a precise understanding of the temporal sequence of events that drive tumor progression remains elusive. In addition to finding that cigarette smoke (CS) impacts the functioning of key pathways with significant roles in redox homeostasis, xenobiotic detoxification, cell cycle control, and endoplasmic reticulum (ER) functioning, our data highlighted a defensive role for the unfolded protein response (UPR) program. The UPR promotes cell survival by reducing the accumulation of aberrantly folded proteins through translation arrest, production of chaperone proteins, and increased degradation. Importance of the UPR in maintaining tissue health is evidenced by the fact that a chronic increase in defective protein structures plays a pathogenic role in diabetes, cardiovascular disease, Alzheimer's and Parkinson's syndromes, and cancer.

**Methods:**

Gene and protein expression changes in CS exposed human cell cultures were monitored by high-density microarrays and Western blot analysis. Tissue arrays containing samples from 110 lung cancers were probed with antibodies to proteins of interest using immunohistochemistry.

**Results:**

We show that: 1) CS induces ER stress and activates components of the UPR; 2) reactive species in CS that promote oxidative stress are primarily responsible for UPR activation; 3) CS exposure results in increased expression of several genes with significant roles in attenuating oxidative stress; and 4) several major UPR regulators are increased either in expression (i.e., BiP and eIF2α) or phosphorylation (i.e., phospho-eIF2α) in a majority of human lung cancers.

**Conclusion:**

These data indicate that chronic ER stress and recruitment of one or more UPR effector arms upon exposure to CS may play a pivotal role in the etiology or progression of lung cancers, and that phospho-eIF2α and BiP may have diagnostic and/or therapeutic potential. Furthermore, we speculate that upregulation of UPR regulators (in particular BiP) may provide a pro-survival advantage by increasing resistance to cytotoxic stresses such as hypoxia and chemotherapeutic drugs, and that UPR induction is a potential mechanism that could be attenuated or reversed resulting in a more efficacious treatment strategy for lung cancer.

## Background

The long lag time between initiation of cigarette smoking and cancer induction (estimated at 25 to 50 pack-years) [1,2] raises several fundamental questions concerning the eventual induction of tobacco-induced diseases for which there is little information: e.g., how does the lung adapt to the chronic assault of many decades of cigarette smoke (CS) exposure, what are the biological sequelae that occur in response to this adaptation and the continuous disruption of normal cellular homeostasis in the lung, and is this adaption a help or hindrance to lung cancer development? Our working hypothesis is that a) tobacco-induced lung cancer is a complex process in which numerous pro-survival cellular systems have important contributory functions that both augment and modify the central role played by tobacco carcinogens and reactive oxygen/nitrogen species, and b) CS temporally shapes the course of lung carcinogenesis through chronic activation, and eventual dysregulation, of normal cellular defense mechanisms. In our published [3-6] and unpublished studies using high-density oligonucleotide arrays and other techniques to define relevant CS-induced alterations in gene/protein expression and function in lung cells, we have attempted to place the impacted genes into biological context by developing a plausible mechanistic model relating disruption of specific cellular circuits to pulmonary disease. Thus, in addition to revealing that CS affects the functioning of several important molecular pathways (e.g., redox homeostasis, detoxification of xenobiotics and cell cycle control), these data highlighted a potential role for the unfolded protein response (UPR) program.

Successful maturation of secretory and membrane proteins in the endoplasmic reticulum (ER) involves proper folding, assembly, and post-translational modification [7]. A wide range of stressful situations (e.g., hypoxia, viral infection, alterations in glycosylation status, disruption of calcium homeostasis, and oxidative stress), can disrupt this maturation process, resulting in the accumulation of unfolded or misfolded proteins and causing ER stress [8]. The ER attempts to attenuate this stress by activating an adaptive set of stress response signaling pathways termed the Unfolded Protein Response (UPR) [8,9]. The primary function of the UPR is to reduce the accumulation of aberrantly folded proteins in the ER and promote cell survival through a transient decrease in protein translation coupled with increases in the ER's capacity to refold and degrade these proteins[10,11]. If this pro-survival response fails to restore homeostatic equilibrium in the ER, a secondary response, triggered in part by the same ER stress sensors that activate the UPR program, promotes apoptosis and cell death. The importance of a properly functioning ER in maintaining cellular and tissue health is clear from the mounting evidence that a chronic increase in defective protein structures coupled with dysregulation within the ER can play a pathogenic role in diabetes, cardiovascular disease, Alzheimer's and Parkinson's syndromes, and cancer [12-14].

An accumulation of misfolded proteins induces the dissociation of the ER-resident master chaperone regulator, BiP/GRP78 (Binding Immunoglobulin Protein/Glucose Response Protein 78), from three ER transmembrane sensor proteins: ATF6 (Activation of Transcription Factor 6), Ire1 (Inositol Requiring Enzyme 1α), and PERK (Protein Kinase R-like ER Kinase) resulting in activation of their respective molecular functions [15,16]. A second mechanism driving activation of these sensor proteins may also involve binding of unfolded protein domains to a peptide-binding groove in both IRE1 and PERK, and possibly ATF6 [17]. Upon experiencing stress the 90 kDa ATF6 protein translocates from the ER to the Golgi where it is proteolytically processed to a functional 50 kDa transcription factor that binds to specific ER stress elements and directs the synthesis of chaperone proteins that mitigate protein misfolding through various mechanisms [18,19]. IRE1 has, in addition to a kinase domain, an endoribonuclease domain that splices an intron from the XBP1 (X-box Binding Protein) mRNA resulting in the synthesis of a transcriptional activator that modulates expression of a number of genes involved in ER homeostasis, DNA damage repair, and redox homeostasis [20-22]. As well as being a transcriptional activator, IRE1 can also mediate the rapid degradation of a specific subset of mRNAs that would interfere with the coordinated reestablishment of normal ER function [23]. In contrast to these pro-survival functions, IRE-1 can also directly regulate both pro- and anti-apoptotic circuits via activation of the stress kinase JNK-1 and mitogen-activated protein kinase ERK-1 [24-26]. Unlike the transcriptionally active ATF6 and XBP1 proteins, PERK, upon release from BiP/GRP78, undergoes autophosphorylation and activation of a kinase function that phosphorylates the alpha subunit of eIF2 (eukaryotic translation initiation factor 2) and a transient repression of global protein synthesis. This temporary decrease in newly synthesized proteins entering the ER provides time to reestablish homeostatic equilibrium and resume normal protein maturation [27,28].

Since habitual U.S. smokers consume an average of 16.6 cigarettes/day [29], and probably inhale somewhere between 120–180 puffs/day of a complex mixture of reactive gases and particulate matter composed of a wide range of entities (e.g., carcinogens and reactive oxygen/nitrogen radicals) that cause both immediate and delayed damage to proteins, lipids, and nucleic acids, it is likely that the lung cell is protected by multiple pro-survival mechanisms, including the UPR. Two recent studies have begun to amass data showing that CS induces elements of the UPR program in lung cells [30,31]. These reports speculate that elements of the UPR signaling pathway are relevant to human smoking-related diseases. However, there is minimal data detailing the impact of CS on all three UPR effector arms in both normal and malignant lung cells. Moreover, there is inconclusive evidence of UPR activation in human lung cancers. Thus, we were interested in determining the impact of CS on the UPR pathway in NHBE lung cells in vitro, and assessing the status of UPR activation in vivo in human lung cancers. We report here that CS exposure induces ER stress and triggers the UPR in both normal and malignant human lung cells. Of greater clinical relevance, however, is that an immunohistochemical analysis of a human lung tumor tissue array containing 110 lung cancer specimens and 10 normal lung tissue controls from a total of 120 patients showed a statistically significant increase in the total levels of phospho-eIF2α, BiP, and eIF2α in lung cancers compared to non-malignant lung cells.

These data implicate dysregulation of the UPR pathway in the pathogenesis of human lung cancers and indicate that phospho-eIF2α and BiP (and possibly eIF2α), may have diagnostic and/or therapeutic potential [32,33]. Furthermore, the activation of the UPR program via eIF2α phosphorylation indicates a previously unknown pathogenic effect of CS and suggests that chronic induction of one or more protein effectors of the UPR pathway may play an etiological role in lung cancer. Finally, the upregulation of several UPR regulators (in particular) in lung cancers may provide a pro-survival advantage by increasing cellular resistance to various cytotoxic stresses such as hypoxia, chemotherapeutic drugs, and immune attack [34-38], especially in tumor cells that have one or more pro-apoptotic pathways disabled, which is a common feature of lung neoplasms [39].

## Methods

### Cell Culture and Smoke Treatment

A549 cells were purchased from American Type Culture Collection (ATCC no. CCL-185, Manassas, VA) and were cultured in Ham's F12K medium with 2 mM L-glutamine adjusted to contain 1.5 g/L sodium bicarbonate (Gibco/Invitrogen, Carlsbad, CA) and supplemented with 10% fetal bovine serum (ATCC). NHBE cells from nonsmoking, nondiabetic donors were purchased from Cambrex Corporation (Walkersville, MD). Cells were cultured in complete Bronchial Epithelial Cell Growth Medium, prepared by supplementing Bronchial Epithelial Basal Medium with retinoic acid, epidermal growth factor, epinephrine, transferrin, T3, insulin, hydrocortisone, antimicrobial agents and bovine pituitary extract by addition of SingleQuots,™ (Cambrex Corporation). All cell cultures were treated before their sixth passage. All incubations were at 37°C in a humidified atmosphere of 5% CO_2 _in air. CS treatment was performed as follows: cells were seeded into 35 mm Petri dishes (Fisher Scientific, Falcon #35-3001, Pittsburg, PA) at a density of 10^5 ^cells/dish and were typically at 70% confluency at the time of exposure to CS. At least three replicate dishes were treated for each condition, and each replicate was analyzed using a separate microarray (i.e., the RNA from the dishes was not pooled). The cell culture medium was replaced with 37°C Dulbecco's PBS (D-PBS) containing calcium and magnesium (Gibco/Invitrogen) for the smoke exposure. The covers were removed from the Petri dishes and they were placed in a smoke exposure chamber designed to deliver a consistent dose of diluted CS. CS was generated under Federal Trade Commission (FTC)[40] smoking conditions (35 ± 0.3 cc puff, one puff every 60 seconds, 2-second puff duration with none of the ventilation holes blocked) using a KC 5 Port Smoker (KC Automation, Richmond, VA), from 2R4F reference research cigarette (designed to represent the average 'lights' cigarette marketed in the U.S. with FTC values of 9.7 mg 'tar' and 0.85 mg nicotine; University of Kentucky, Louisville, KY) using FTC machine smoking conditions, or two leading commercially available U.S. cigarette brands representative of either the 'lights' (FTC values of 11 mg 'tar' and 0.8 mg nicotine) or 'full-flavor' (FTC values of 15 mg 'tar' and 1.1 mg nicotine) styles of cigarettes. Cigarettes were smoked to within 3 mm of the filter tip. All cigarettes had been equilibrated at 23.9°C ± 1.1°C and 60% ± 2% relative humidity for a minimum of 24 hours and a maximum of 14 days. The smoke exposure chamber was designed to deliver smoke uniformly diluted with 5% CO_2 _in air and passed through the cell exposure chamber at a constant flow rate of 500 cc/min. Briefly, each 35 cc puff was first drawn into a 250 cc round chamber containing 5% CO_2 _in air and mixed via a stir bar. The standard smoke dilution used in most of our experiments was 35 cc delivered over 1 min in a 250 cc or 500 cc volume, and the intensity of exposure was varied by varying the length of time the cells spent in the exposure chamber (typically 15 min or 20 min). The time and distance that the smoke traveled from the end of the cigarette to the exposure chamber was minimized by using the shortest lengths of tubing possible between the parts of the apparatus. Mock-exposed cells were treated under identical conditions as the exposed cells except for the absence of a cigarette in the smoking port. Following treatment or mock treatment, the D-PBS covering the cells was aspirated and replaced with 1 ml per chamber of fresh culture medium at 37°C. The cells were placed in the 37°C, 5% CO_2 _incubator for the times indicated.

### Thapsigargin, tunicamycin, and dithiothreitol treatment

UPR-stimulating reagents thapsigargin, tunicamycin, and dithiothreitol (DTT) were obtained from Sigma, St. Louis, MO. Treatment conditions used in our experiments were 1 uM thapsigargin, 10 μg/mL tunicamycin, or 2 mM DTT added to the cell culture medium in DMSO (thapsigargin and tunicamycin) or water (DTT), and the cells were placed in the 37°C, 5% CO_2 _incubator for the times indicated. In experiments where thapsigargin or tunicamycin treatment followed smoke exposure, the PBS was removed from the cell cultures immediately after smoke exposure and replaced with fresh medium containing the thapsigargin or tunicamycin, and placed in the 37°C, 5% CO_2 _incubator for the times indicated.

### Microarray analysis

Cells were harvested for total RNA extraction after either mock or smoke treatment. The medium was aspirated and the dishes were rinsed twice with 1 mL prewarmed PBS per dish. After the second rinse, 350 ul of Buffer RLT (Qiagen Inc, Valencia, CA) was added per dish. NHBE cell lysates were homogenized using a QIAshredder spin column and RNA extracted using Qiagen RNeasy spin columns according to the manufacturer's protocol (Qiagen). The eluted RNA was frozen and stored at -80°C. Microarray data was generated at Expression Analysis (Durham, NC). RNA integrity was assessed using capillary gel electrophoresis (Agilent BioAnalyzer, Agilent Technologies, Palo Alto, CA) to determine the ratio of 28s:18s rRNA in each sample. The RNA quality of all samples was extremely high with no RNA Integrity Number (RIN) being less than 9.5 out of 10.0 RIN units (a detailed explanation of RIN units can be found at ). Two micrograms of total RNA was reverse transcribed into double stranded cDNA using an oligo(dT)/T7 promotor chimeric primer, and in vitro transcribed using reagents provided by Affymetrix (Santa Clara, CA). Fragmented biotin-labeled cRNA at a concentration of 50 ng/ul was hybridized to Affymetrix Human Genome U133 Plus 2.0 GeneChip^® ^expression arrays according to the manufacturer's recommendations for a minimum of sixteen hours. Post-hybridization washing and staining was performed on the GeneChip^® ^Fluidics Station 450 and arrays were scanned with the GeneChip^® ^Scanner 3000 7G, under the control of the Affymetrix GeneChip^® ^Operating Software (GCOS). Preliminary expression data analysis was performed by GCOS. Two-Group Comparisons with Permutation Analysis for Differential Expression (PADE) was used to determine differential gene expression between CS-exposed and mock-exposed groups at each time point, each group consisting of multiple arrays. PADE analysis provides statistically valid summary measures including false discovery rates along with transcript sets that are typically much more useful and indicative of differential expression between groups than using techniques such as p-values alone, corrected p-values, or p-values with fold change estimates. A complete description of this type of analysis can be found at the Expression Analysis website: . A false discovery rate of 1% and a fold-change level of 1.5 were used as cutoff values. The full microarray data sets have been deposited according to MIAME standards on the NCBI GEO (Gene Expression Omnibus) website:  under the GEO accession numbers GSE10700 and GSE10718.

### PCR analysis of XBP1 splicing

RNA was harvested using the Qiagen RNeasy mini kit according to manufacturer's instructions immediately. First-strand cDNA synthesis was performed with the High Capacity cDNA Reverse Transcription kit for RT-PCR (Applied Biosystems, Foster City, CA). To amplify XBP1 mRNA (NM_005080), PCR was performed for 35 cycles (95°C for 30s; 58°C for 30s; 72°C for 1 min) using the PCR primers 5'-CTG GAA AGC AAG TGG TAG A-3' and 5'-CTG GGT CCT TCT GGG TAG AC-3' with AmpliTag Cold DNA polymerase (#N808-0241; Applied Biosystems). Fragments representing spliced and unspliced XBP1 (398 bp and 424 bp, respectively) were visualized on 2% agarose gels with ethidium bromide staining. NIH ImageJ software (, [41]) was used to quantify gel band intensities.

### Transfection of A549 Cells with ATF6 plasmid

A549 cells were seeded in 35 mm culture dishes at 1.2 × 10^5 ^cells per dish. At 24 hours after the seeding, cells were transfected with ATF6 expression plasmid ATF6/pCMV6-XL5 (OriGene Technologies, Inc., Rockville MD, Catalog # SC115551). Transfection was performed with Lipofectamine LTX (Invitrogen catalog # 15338-100) according to the manufacture's recommendation. Briefly, 2 μg of the plasmid DNA was diluted in 400 ul serum-free medium and mixed with PLUS Reagent (Invitrogen catalog #11514-015) at 1:1 ratio (DNA μg: PLUS vol. in μl). The mixture was incubated at room temperature for 5 min. Lipofectamine LTX was then added to the DNA/PLUS mixture and incubated at room temperature for an additional 25 min. At the end of the incubation, the original growth medium was removed and 2 ml of fresh pre-warmed growth medium was added to each dish. The DNA/PLUS/Lipofectamine complex was immediately added to the appropriate culture dishes, gently mixed and returned to the incubator for another 6 hours after which the medium was replaced again with fresh pre-warmed complete growth medium and the cells were returned to the incubator until smoke exposure or DTT treatment 24 hours post-transfection. Control cells were transfected with pCMV6-XL5 plasmid using the same procedure as above.

### Transfection of A549 cells with PERK siRNA

A549 cells were seeded in 100 mm^2 ^culture dishes at 9 × 10^5 ^cells per dish. At 20 hours after seeding, cells were transfected with PERK siRNA oligos (Dharmacon, Lafayette, CO, catalog # L-004883-00) or non-target siRNA oligos (Dharmacon, catalog # D-001810-10) using DharmaFECT1 transfection reagent (Dharmacon catalog # T-2001-02). Briefly, 21 μl of DharmaFECT1 was diluted in 679 μl of serum-free medium and was incubated at room temperature for 5 minutes. In a separate sterile tube, 70 μl of siRNA oligos (20 μM stock) was mixed with 630 μl of serum-free medium and incubated at room temperature for 5 minutes. The diluted DharmaFECT1 and diluted siRNA oligos were then mixed together and incubated at room temperature for another 20 minutes. At the end of the incubation period, 12.6 ml of complete growth medium was added to the mixture and 14 ml of this final mixture was dispensed to each of the 100 mm^2 ^dish after the original growth medium was removed by aspiration. The dishes were returned to the incubator for another 6 hours before the oligo-DharmaFECT1 containing medium was replaced with fresh prewarmed complete medium. The culture was then allowed to grow in the incubator for another 20 hours when the cells were trypsinized and reseeded in 35 mm^2 ^dishes at 1 × 10^5 ^cells per dish. The 35 mm^2 ^dishes were returned to the incubator and the cells were allowed to grow for another 48 hours before smoke exposure or thapsigargin treatment.

### Cell Lysis for Western Blots

Following treatment, the culture medium was aspirated, cell monolayers were washed twice with cold D-PBS, 2 ml/dish, and 1 ml (per 10^6 ^cells) of RIPA cell lysis buffer (Pierce, Rockford, IL) containing protease and phosphatase inhibitors was added to the monolayer. Cells were dislodged using a cell scraper, transferred to an eppendorf tube, vigorously pipetted, and left on ice for 25 minutes to allow complete lysis. The cell lysates were centrifuged at 10,000 × g for 25 minutes and the supernatants transferred to fresh eppendorf tubes. All manipulations were done at 4°C. Protein concentration of the lysates were determined against a commercially available protein standard using the BioRad protein assay kit (both from Bio-Rad Laboratories, Hercules, CA)

### NHBE cell nuclear fractionation for ATF4 detection

NHBE cells (3^rd ^passage) were seeded at 6 × 10^5 ^cells per 100 mm^2 ^dish two days prior to the smoke exposure. On the day of smoke exposure cells were trypsinized and counted at 4 hours and 7 hours post-CS exposure or after 6 hours of incubation in media containing 1 μM thapsigargin. The total cell numbers were calculated and used for adjusting buffer volumes during cell compartment fractionation. The cellular compartment fractionation was carried out using the Qproteome Cell Compartment Kit (Qiagen catalog # 37502) according to manufacturer's instructions.

### Western Blotting

Protein lysates were run on Criterion Tris-precast polyacrylamide gels (Bio-Rad Laboratories) with SeeBlue Plus2 and Magic Mark protein standards (both from Invitrogen, Carlsbad, CA) and transferred to a PDVF membrane using a Criterion Blotter apparatus (Bio-Rad Laboratories) for ~40 min at room temperature using a transfer buffer containing 10% methanol (Bio-Rad Laboratories). Primary antibodies were from the following sources: Cell Signaling Technology, Danvers, MA (eIF2α cat#9722, phospho-eIF2α cat#3597, BiP cat#3183, Lamin A/C cat#2032 & α-tubulin cat#2144), GeneTex, Inc., San Antonio, TX (eIF2A (phospho S52)Cat # GTX24837), Santa Cruz Biotechnology Inc., Santa Cruz, CA (ATF3 cat#sc-188, ATF4 cat#sc-200), Abcam Inc., Cambridge, MA (GAPDH cat#ab8245, α-tubulin cat#24246), Imgenex, San Diego, CA (ATF6 cat#IMG-273). Membranes were blocked with 5% BSA (for eIF2α, phospho-eIF2α, ATF6, BiP and α-tubulin), 5% BLOTTO (Pierce) for ATF3 and ATF4, and T-20 (Pierce) for GAPDH for 1 hour at room temperature, and then washed with TBST (Bio-Rad) for 3 × 10 min. Membranes were placed in 5% BSA, 5% Blotto or T-20 buffer solution containing the primary antibody (1:1000 dilution for anti-eIF2α, anti-phospho-eIF2α, BiP, and Lamin A/C, 1:200 dilution for ATF3 and ATF4, 1:500 for ATF6, 1:2000 for α-tubulin, and 1:40,000 for GAPDH) and incubated at 4°C overnight with gentle shaking, followed by washes in TBST for 3 × 10 min. Membranes were then placed in 5% BSA, 5% Blotto or T-20 solution containing anti-rabbit-HRP, or anti-mouse-HRP secondary antibody (Cell Signaling Technology) at a 1:2000 dilution and incubated for 1 hour at room temperature with gentle shaking, after which they were washed with TBST for 3 × 10 min. Blots were developed using Western Lightning chemiluminescence reagent (Perkin-Elmer, Boston, MA) according to manufacturer's instructions. NIH ImageJ software (, [41]) was used to quantify gel band intensities.

### Tissue Microarrays (TMAs)

Commercial Cancer/Normal Lung Tissue arrays were acquired from Asterand (Detroit, MI). Arrays were constructed from formalin-fixed paraffin-embedded tissues representing 120 normal and lung cancer cases. Each tissue microarray contained 10 normal cases and 110 tumor cases from a broad range of lung cancer types. Every tissue sample was reviewed by a board certified pathologist to confirm tissue type, diagnosis and optimal region for coring before use. Cores of 0.6 diameter were removed from each selected sample and placed in a paraffin block using a Beecher Manual Array instrument. Each case was represented in triplicate for a total of 360 cores. An H&E slide of each array post construction was completed as a quality control measure. Table 1 lists the pathological characteristics of these 120 formalin-fixed, paraffin-embedded archival tissues from patients.

**Table 1:** Clinicopathological characteristics of the assessed lung cancers.

Clinical Variable	Subcategory	Number of Samples
Age at excision: 32–76 (mean = 60.2 yrs)		
Small Cell Carcinoma	Small cell carcinoma	5
Non-Small Cell Carcinoma		93
	Adenocarcinoma	39
	Adenosquamous carcinoma	12
	Squamous cell carcinoma	37
	Large cell carcinoma	4
	Bronchioloalveolar carcinoma	1
Mixed Carcinoma Types		12
	Carcinoid tumor	2
	Carcinoma	3
	Clear cell carcinoma	1
	Undifferentiated carcinoma	2
	Malignant mesothelioma	4

### Antibodies and immunohistochemical analysis

Identical TMAs were immunostained with one of the following antibodies: a) a rabbit monoclonal anti-BiP antibody (Cell Signaling Technology, Danvers MA, cat. # 3177) recognizing BiP (C50B12) from rabbits immunized with a synthetic peptide derived from the sequence around Gly584 of human BiP (used at 1:200 dilution and a concentration of 1 μg/ml); b) a mouse monoclonal anti-EIF2 antibody (Cell Signaling Technology cat. #2103) recognizing eIF2α (L57A5) from mice immunized with purified recombinant human eIF2α (used at 1:50 dilution and a concentration of 0.1 μg/ml); c) a rabbit monoclonal anti-phospho-EIF2 antibody (Cell Signaling Technology cat.# 3597) recognizing Ser51 (used at 1:50 dilution and a concentration of 1 μg/ml); d) an irrelevant rabbit IgG antibody control (Jackson ImmunoResearch, West Grove, PA) (used at a concentration of 1 μg/ml). Prior to antibody staining, slides were deparaffinized in 3 changes of xylene and rehydrated in graded ethanol. All slides were subjected to antigen unmasking in 10 mM citrate buffer, pH 6.0, quenching in 3% H_2_O_2 _and blocking prior to incubation with primary antibodies overnight at 4°C. Following incubation with the primary antibodies, detection was performed using the ABC Elite Kit (Vector Laboratories, Burlingame, CA) with either a biotinylated goat anti-rabbit secondary antibody or a biotinylated goat anti-mouse secondary antibody (Jackson ImmunoResearch). Both eIF2α mouse monoclonal and BiP rabbit monoclonal antibodies from Cell Signaling Technology had previously been validated at the company using human breast carcinoma samples and multi-tissue arrays. The sequence specificity of the BiP antibody was verified using peptide blocking. The EIF2 ?antibody was raised against a fusion protein and thus was not validated with peptide blocking. The antibody did give the expected expression and subcellular localization. Human breast carcinoma slides used as a control were obtained from Newcomer Supply (Madison, WI). The anti-phospho-eIF2α antibody has been validated at Cell Signaling Technology using 3T3 -/+ thapsigargin cell pellets, human breast carcinoma samples and a multi-tissue array. Phospho-specificity of the antibody was confirmed using lambda phosphatase pre-treatment of tissue sections, and sequence specificity was verified using peptide blocking.

### Clinicopathological variables

The following variables were correlated with protein expression: a) tumor stage, b) age at tumor excision, c) gender, d) ethnicity, e) smoking status (i.e., never user, occasional user, former user, current user); f) number of cigarettes per day, and g) smoking duration. Staging adhered to the AJCC pTNM (tumor-node-metastasis) staging system and included both clinical and histological data.

### Staining criteria and statistical analyses

Tumor cells and non-tumor cells were scored separately for percent immunohistochemical reactivity and staining intensity for BiP, eIF2α, and phospho-eIF2α expression. The percentage of cells staining positive was assigned one of the following grades: (0) < 5%; (1) 5–25%; (2) 25–50%; (3) 50–75%; (4) > 75%. Staining intensity was independently graded on the following scale: (0) none; (1) weak; (2) moderate; (3) strong; (4) intense. In order to more accurately reflect the synergistic contribution of both criteria to overall protein expression, the product of the percentage and intensity scores was used to generate an immunohistochemical staining index (ISI) for each cellular component (i.e., tumor and non-tumor) of an individual tissue specimen [42,43]. Staining was independently assessed by two pathologists. Measurements were made in triplicate. For purposes of statistical analysis, triplicate measurements were represented by their mean. To compare mean ISI levels between each carcinoma diagnostic group with the normal group, one-way analyses of variance followed by Dunnett's tests were used. To compare the ISI levels in the tumor and normal cell compartments of each specimen, repeated measures analyses of variance followed by Tukey's tests were used. A result was considered statistically significant if the resulting *P *value was less than or equal to 0.05.

## Results

### Generation of a CS Gene Expression 'Signature' in NHBE cells

Despite the fact that commercial brands of cigarettes sold in the U.S. differ in terms of tobacco blends, flavorings, general construction attributes, and 'tar'/nicotine yields, epidemiological studies indicate essentially no significant difference in lung cancer risk among long-term smokers of different cigarette brands or styles (i.e., ultralights, lights, or full-flavor) [44-48]. The obvious conclusion from these data is that the chemical composition and overall toxicity of all brands and styles of currently available U.S. cigarettes are generally comparable. Therefore, it is reasonable to assume that these cigarettes also have a similar molecular impact on cells and tissues, at least with respect to those genes/proteins relevant to disease. In order to link one or more of these genes/proteins to the various pathological states observed in smokers a logical first step would be to first generate a generic CS-specific gene or protein expression 'signature' that is reflective of cigarettes regardless of brand or style. Consequently, NHBE cells were exposed in vitro to whole smoke from three different cigarettes: the 2R4F Kentucky Reference Cigarette which is a 'lights' type cigarette (FTC values of 9.7 mg 'tar'/0.85 mg nicotine), and two leading U.S. commercial brands representative of a 'full flavor' type (Brand A: FTC values of 15 mg 'tar'/1.1 mg nicotine) and a 'lights' type (Brand B: FTC values of 11 mg 'tar'/0.8 mg nicotine).

Using the exposure chamber as described in the Methods section, NHBE cells were exposed to air or CS generated under FTC smoking conditions for 15 min, which for the CS-exposed samples resulted in an average total particulate matter (TPM) deposition per cigarette of 3.14 μg/cm^2 ^(Brand A, 'full-flavor' type), 2.70 μg/cm^2 ^(Brand B, 'lights' type), and 2.56 μg/cm^2 ^(2R4F reference cigarette). In vivo studies measuring particulate matter distribution in the human lung have estimated that smoking a single 10 mg 'tar' cigarette results in the deposition of between 0.74 to 1.94 μg of particulate matter/cm^2 ^[49,50]. Subsequent to the 15 min treatment with either CS or air, the cells were subjected to a post-exposure washout period (incubation in fresh media) for 2, 4, or 24 h after which the cells were lysed and RNA extracted for microarray analysis. Viability of CS-treated cells was typically greater than 50% that of air-treated controls when assessed by trypan blue exclusion.

Gene expression data from the 'full-flavor' Brand A, 'lights' Brand B, and the 2R4F reference cigarette at each of the three post-exposure time points was compared to data from the corresponding air-exposed control samples to determine differential gene expression patterns resulting from CS exposure. Perturbations in gene expression common to all three cigarette variants were taken to represent the common CS signature for each time point. Specific gene lists comprising CS signatures for 2 h, 4 h and 24 h are provided in additional file 1 – **Supplemental Table S1: Cigarette smoke signature genes**. The high-stringency inclusion criteria used to generate these lists (see Methods section) greatly increases the probability that the genes will be highly relevant to tobacco-related impact. To elucidate the major biological processes represented by the CS signatures at each time point, the gene lists were subjected to Functional Annotation Enrichment Analysis using statistical tools available at the newly-updated Database for Annotation Visualization and Integrated Discovery (DAVID) web site . CS signature gene lists were uploaded to DAVID where they were functionally annotated and clustered. We used Gene Ontology (GO) annotations to explore biological significance of the gene lists rather than curated pathway data such as KEGG pathways because more thorough annotation information is available for GO classes than for KEGG pathway involvement. For example, 72% of the genes in a test list had GO Biological Process annotation as opposed to only 21% represented in KEGG pathways. Biological processes significantly over-represented in the uploaded gene lists were identified and clustered according to GO Biological Process categories. The mean *p*-value for each cluster was determined and used to list the clusters in order of significance (additional file 2 – **Supplemental Table S2: Functional annotation clustering of cigarette smoke signature genes**). Examination of the most significant clusters (*p*-value < 0.01) for the 4 h upregulated CS signature revealed several processes characteristic of exposure to xenobiotics such as response to stress, response to chemical stimulus, and response to abiotic stimulus. Notably, some of the lowest *p*-values at 4 h are the GO categories 'response to unfolded proteins' and 'protein folding'. Based on this observation, and because it is increasingly clear that the UPR can play a pathogenic chronic role in a wide range of diseases including cancer [12-14,51] we decided to further assess the effects of CS treatment on UPR pathway components in lung cells.

### CS treatment induces the phosphorylation of eIF2α by PERK in normal human lung cells

**Figure 1:**
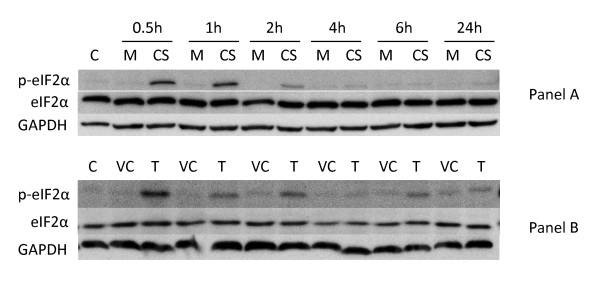
**Induction of eIF2α phosphorylation in NHBE cells by cigarette smoke and thapsigargin**. **Panel A: **NHBE cells were exposed to air (M = mock treatment) or 2R4F cigarette smoke with 35 cc puffs diluted in 250 cc air (CS = smoke treatment) for 20 minutes. Cells were then placed in fresh medium and returned to the incubator for the time periods specified. Western blots of whole cell lysates were probed with antibodies to phosphorylated eIF2α, eIF2α, and GAPDH as a loading control. C = untreated control. **Panel B: **NHBE cells were treated with either 1 uM thapsigargin in DMSO (T), or DMSO (VC = vehicle control) for the times specified. Western blots of whole cell lysates were probed with antibodies to phosphorylated eIF2α, eIF2α, and GAPDH. C = untreated control.

The canonical pathway for ER stress activation of the UPR begins with the dissociation of BiP from PERK, resulting in PERK autophosphorylation. Upon activation, PERK phosphorylates eIF2α at serine-51, a key step in the process which inhibits translation by preventing the recruitment of the initiator methionyl tRNA to ribosomes (for review see [52]). The suppression of protein translation via phosphorylation of both PERK and eIF2α in rodent cells in response to treatment with cigarette smoke-bubbled PBS has recently been demonstrated [31]. However, in order to investigate PERK-dependent eIF2α phosphorylation in diploid normal human bronchial epithelial (NHBE) lung cells as well as malignant human lung cells (A549) treated with a relatively brief exposure to whole smoke, we assessed the response of cells exposed to CS from 2R4F reference cigarettes as well as 'full-flavor' Brand A, 'lights' Brand B for the transient phosphorylation of eIF2α. Figure 1, Panel A shows that exposure of NHBE to CS from 2R4F reference cigarettes resulted in the rapid phosphorylation of eIF2α within 30 minutes, which peaked between 30–60 minutes and diminished to mock-treated control levels by 4 hours. Equivalent results were seen for A549 cells (data not shown). This is a similar time course to that seen after exposure to thapsigargin (1 μM), a potent inducer of ER stress and eIF2α phosphorylation (Figure 1, Panel B). Figures 1A and 1B also show that during the time period studied (0–4 hours after CS exposure) there were no significant changes in protein levels of total eIF2α after exposure to either CS or thapsigargin. Similar to the results observed with 2R4F cigarettes, CS generated from the 'full flavor' Brand A and 'lights' Brand B cigarettes also induced the rapid eIF2α phosphorylation with essentially the same time course as that observed for 2R4F (data not shown), suggesting that activation of the UPR pathway is common to all types of cigarettes.

**Figure 2:**
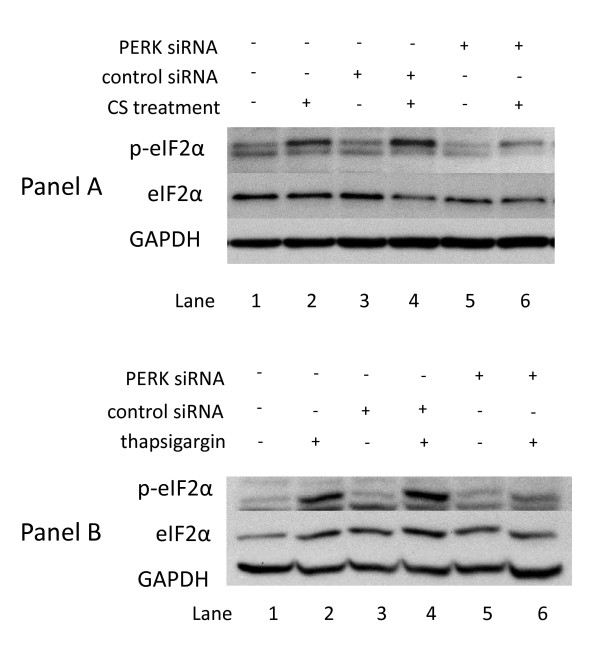
**Dependence of eIF2α phosphorylation on PERK in A549 cells treated with cigarette smoke (and thapsigargin**. **Panel A: **A549 cells were transfected with control siRNA (lanes 3 and 4) or PERK siRNA (lanes 5 and 6) as described in Methods. 24 h post-tranfection the cells were exposed to air (lanes 1, 3, and 5) or 2R4F cigarette smoke with 35 cc puffs diluted in 250 cc air (lanes 2,4, and 6) for 20 minutes. Cells were then placed in fresh medium and returned to the incubator for the time periods specified. Western blots of whole cell lysates were probed with antibodies to phosphorylated eIF2α, eIF2α, and GAPDH as a loading control. **Panel B: **A549 cells were transfected with control siRNA (lanes 3 and 4) or PERK siRNA (lanes 5 and 6) as described in Methods. 24 h post-tranfection the cells were treated with either 1 uM thapsigargin in DMSO (lanes 2,4, and 6) or DMSO (lanes 1, 3, and 5) for the times specified.

In addition to PERK, three other kinases capable of phosphorylating eIF2α (PKR, HRI, and GCN2, reviewed in reference [52]) have been identified in mammals. Although current understanding indicates that these other kinases are regulated by different stress stimuli [52], their catalytic domains are homologous. Thus, phosphorylation of eIF2α by any of these four kinases results in similar downstream events, including translation attenuation and activation of transcriptional programs that augment the cell's ability to cope with problematic conditions such as the accumulation of unfolded proteins, amino acid deprivation, or oxidative stress [52]. However, PERK-induced activation of eIF2α is believed to occur exclusively upon the induction of ER stress [11]. Consequently, in order to show that CS treatment induces ER stress, it is important to demonstrate the dependence of CS-induced phosphorylation of eIF2α phosphorylation on PERK. Thus, A549 cells were transiently transfected with a plasmid containing siRNA designed to silence PERK, and then exposed to CS or to thapsigargin as an activation control. As shown in Figure 2, PERK siRNA transfected A549 cells show notably reduced phosphorylation of eIF2α when treated with CS (Panel A) or thapsigargin (Panel B), indicating that PERK is the major effector of CS-induced phosphorylation of eIF2α in both these systems. Total eIF2α levels remained constant in all samples regardless of treatment conditions.

### Effect of CS on ATF4 levels and expression of ATF4-dependent genes

In addition to transiently suppressing protein translation during ER stress, a second function of PERK phosphorylation of eIF2α is the upregulation of genes integral to the UPR pro-survival response that promote protein folding and redox homeostasis [53]. For example, eIF2α phosphorylation promotes the induction of activating transcription factor 4 (ATF4) through a mechanism that relies upon unique features in its 5' untranslated region [54]. ATF4 accumulates in the nucleus and upregulates genes that attempt to adapt the cell to ER stress such as activating transcription factor 3 (ATF3), a member of the ATF/CREB family of basic-region leucine zipper (bZIP) proteins [55,56], or the pro-apoptotic transcription factor CCAAT/enhancer binding protein-homologous protein (CHOP) [57]. CHOP and ATF3 in turn coordinate the upregulation of GADD34 which provides a feedback inhibitory effect on UPR [58].

**Figure 3:**
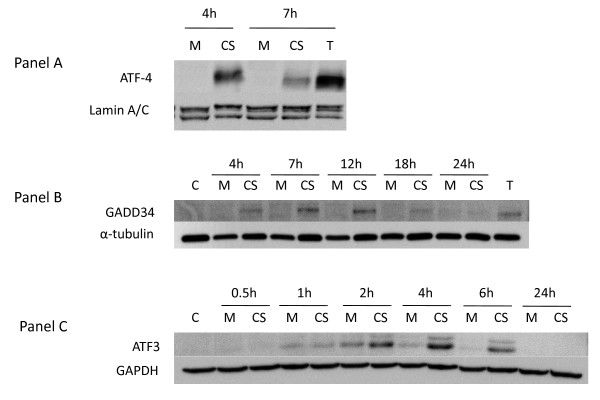
**Effect of CS on ATF4 pathway**. **Panel A: **NHBE cells were exposed to air (M = mock treatment) or 2R4F cigarette smoke with 35 cc puffs diluted in 250 cc air (CS = smoke treatment) for 20 minutes. Cells were then placed in fresh medium and returned to the incubator for the time periods specified. Nuclear extracts were prepared as described in the Methods section. Western blots of nuclear fractions were probed with antibodies to ATF4 and Lamin A/C, a nuclear antigen, as a loading control. **Panel B: **NHBE cells were exposed to air (M = mock treatment) or 2R4F cigarette smoke with 35 cc puffs diluted in 250 cc air (CS = smoke treatment) for 20 minutes. Cells were then placed in fresh medium and returned to the incubator for the time periods specified. Western blots of whole cell lysates were probed with antibodies to GADD34 and α-tubulin as a loading control. **Panel C:**A549 cells were exposed to air (M = mock treatment) or 2R4F cigarette smoke (CS = smoke treatment) for 20 minutes with 35 cc puffs diluted in 250 cc air. Cells were then placed in fresh medium and returned to the incubator for the time periods specified. Western blots of whole cell lysates were probed with antibodies to ATF3 and GAPDH.

Figure 3 (Panel A) shows an increase in the level of nuclear ATF4 in NHBE cells in response to CS treatment at 4 hrs post-exposure which diminishes by 7 hrs. In addition, Figure 3 shows that both GADD34 (Panel B) and ATF3 (Panel C) protein levels also increase in CS-treated cells. ATF3-induced expression has a similar time-course as ATF4 (i.e., peaking around 4 hrs post-exposure) while peak GADD34 expression occurs between 7–12 hrs post-exposure. Consistent with this protein data, the array profiles of cigarette smoke signature genes shown in Additional file 1 – **Supplemental Table S1 **shows that the mRNA transcripts for CHOP (also denoted DDIT3), ATF3, and GADD34 (also denoted PPPIR15A) are upregulated by CS treatment.

### Effect of CS exposure on the ATF6 branch of UPR in human lung cells

**Figure 4:**
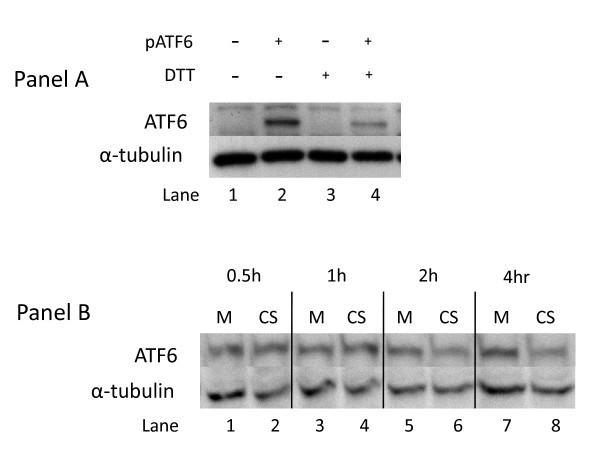
**Effect of CS on proteolytic cleavage of ATF6**. **Panel A: **A549 cells were transfected with a plasmid expressing ATF6 (lanes 2 and 4, indicated by +), or a control plasmid (lanes 1 and 3), then treated with 2 mM DTT for 1 h (lanes 3 and 4) or left untreated (lanes 1 and 2). Western blots of whole cell lysates were probed with antibodies to ATF6 and α-tubulin. **Panel B: **All lanes show lysates from A549 cells transfected with a plasmid expressing ATF6. Cells were exposed to air (M = mock treatment) or 2R4F cigarette smoke (CS = smoke treatment) for 20 minutes with 35 cc puffs diluted in 250 cc air. Cells were then placed in fresh medium and returned to the incubator for the time periods specified. Western blots of whole cell lysates were probed with antibodies to ATF6 and α-tubulin. Gel bands were quantified as described in the Methods section. CS treatment resulted in a 23% and 26% decrease in ATF6 90 kDa protein at 2 and 4 h respectively (lane 5 compared to lane 6, lane 7 compared to lane 8).

**Figure 5:**
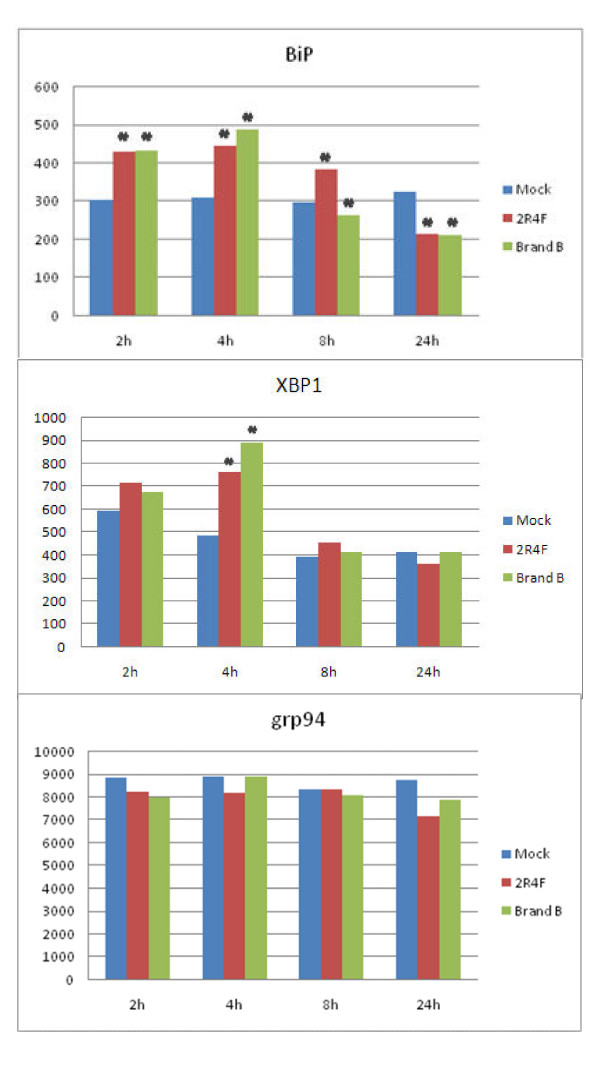
**Expression of BiP, XBP1, and grp94 in CS-treated human lung cells**. NHBE cells were exposed to air (Mock), 2R4F cigarette smoke with 35 cc puffs diluted in 500 cc air (2R4F), or Brand B cigarette smoke with 35 cc puffs diluted in 500 cc air for 15 minutes. Cells were then placed in fresh medium and returned to the incubator for the time periods specified prior to RNA extraction. Four separate microarray representing four separate samples were used to analyze each condition. Mean intensity values are shown. * = *p *< 0.01 when compared to Mock.

In addition to PERK, a second UPR effector arm is activated by proteolytic cleavage of the 90 kDa ATF6 protein and release of a functional 50 kDa transcription factor. Our initial analysis indicated that the basal level of ATF6 expression in A549 cells was below detectable levels (data not shown). Thus, in order to assess the effect of CS on ATF6 cleavage we constructed a full length ATF6/pCMV6 expression plasmid and transfected it into A549 cells which were then exposed to CS. Since robust detection of the 50 kDa end product is difficult as it is unstable and rapidly degraded [59], we monitored the disappearance of the 90 kDa ATF6 protein to assess its activation. Figure 4 (Panel A) shows that treatment of ATF6-transfected A549 cells with DTT, a potent inducer of ER-stress and the UPR due to its disruption of intramolecular disulfide bonds, results in the disappearance of the 90 kDa protein by 1 hr. Similarly, Figure 4 (Panel B) shows that treatment of ATF6-transfected A549 cells with CS also results in disappearance of ATF6 starting at 2 h post-CS exposure and continuing to at least 4 h post-exposure. The reduction in the 90 kDa ATF6 protein could also be due to increased degradation or reduced synthesis. However, when the array data was assessed to determine changes in the levels of three genes whose transcription is dependent on activated ATF6 (i.e., BiP, XBP1, and grp94) [60], we found that both XBP1 and BiP transcript levels are elevated in response to CS treatment (Figure 5). Thus, these data support the conclusion that CS-treatment activates the ATF6-signaling pathway.

### Effect of CS exposure on the expression of BiP in human lung cells

**Figure 6:**
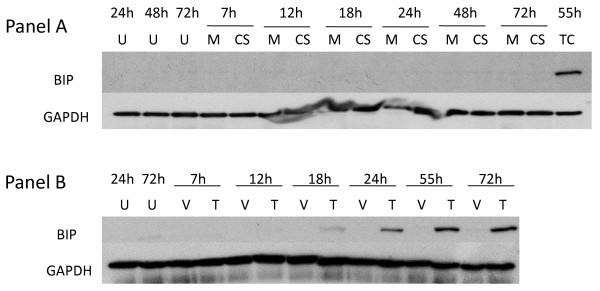
**Effect of thapsigargin and cigarette smoke on expression of BiP**. **Panel A: **A549 cells were treated with 1 mM thapsigargin for the time periods specified. U = untreated cell control; V = vehicle (DMSO) control; T = thapsigargin. **Panel B: **A549 cells were cells were exposed to air (M = mock treatment) or 2R4F cigarette smoke (CS = smoke treatment) for 20 minutes (35 cc puffs were diluted in 250 cc air), after which the cells were placed in fresh medium and returned to the incubator for the time periods specified. Western blots of whole cell lysates were probed with antibodies to BiP and GAPDH. TC = thapsigargin treated cells as a positive internal control for Panel B.

**Figure 7:**
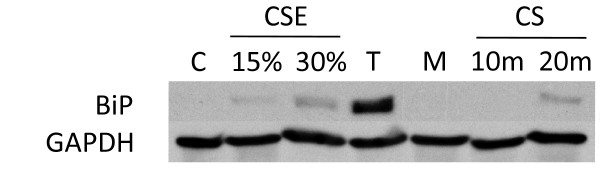
**BiP expression in normal human lung cells treated with CS long term treatment**. NHBE cells were exposed to either 15% or 30% 2R4F cigarette smoke extract (CSE) prepared as described in (ref), or exposed to 2R4F cigarette smoke (CS) for 10 or 20 minutes (35 cc puffs were diluted in 250 cc air), after which the cells were immediately returned to the incubator without a media change for the time periods specified. C = untreated control.

The transient accumulation of misfolded proteins, a key indicator of ER stress, results in the dissociation of the ER-resident master chaperone regulator BiP from all three ER resident sensors (i.e., PERK, IRE1 and ATF6) triggering their activation and subsequent induction of various functions of the UPR that provide the cell with a mosaic of options to counter ER stress and reestablish homeostasis [61,62]. For example, when ER stress becomes chronic, one pro-survival mechanism is to upregulate the master regulator, BiP, which allows the cell to endure an increased level of unfolded proteins and forestall apoptosis [63,64]. In one recent publication, Hengstermann and Muller [31] saw a modest increase in BiP RNA levels in mouse cells after 4 h continuous treatment with PBS into which CS was previously bubbled [31]. A second report by Kelsen et al, [30] observed increased BiP protein levels in virally transformed human bronchial epithelial cells after 24 h continuous treatment with 15% cigarette smoke extract. In contrast to these studies, our results show that when A549 cells (Figure 6, Panel A) or NHBE cells (data not shown) were briefly exposed to freshly generated whole CS for 20 min, Western blot analysis showed no increase of BiP protein levels up to 72 h post-exposure. Control cells treated with thapsigargin show strong induction of BiP by 24 h. We speculated that this apparent discrepancy in BiP expression could be due to the fact that we exposed short-term cultures of untransformed NHBE cells (as opposed to rodent cells or virally-transformed bronchial cells). Alternatively, since there are significant differences between our exposure parameters and those detailed in these two recent reports, a more likely explanation may be that the brief CS treatment protocol (20 min followed by washout and addition of fresh medium) used in our experiments resulted in an abbreviated UPR response that fails to trigger BiP upregulation. Since BiP induction is a relatively late event in the UPR signaling cascade, differing stress-inducing exposure conditions can lead to short term perturbations without durable long-term changes [12]. To test this idea, we prepared CS-bubbled media (CSE) according to the procedure used by Hengstermann and Muller [31] and treated NHBE cells for 24 h in 15% or 30% CSE. Moreover, instead of our usual procedure, we also exposed NHBE cells to CS for 20 min, except that after this treatment, the cells were not placed in fresh media but were incubated in the CS-containing medium for another 24 hrs. We observed that both of these much longer exposure procedures did result in the upregulation of BiP, although not to the degree seen with the more potent ER-stress inducer thapsigargin (Figure 7). We note that when either A549 or NHBE cells are treated with 1 μM thapsigargin continuously for 72 h, BiP expression is still only seen after 24 hours post-treatment, indicating that BiP induction is a much later consequence of ER stress than is eIF2α phosphorylation in these cells (see Figure 6, Panel B). Consequently, these data, while agreeing with previous reports showing that BiP expression is induced by CS [30,31], clearly show that BiP induction can be modulated depending on level and duration of stress-inducing conditions. It has been shown that different cell types and different stress conditions can selectively activate one or more of the ER sensors [65]. Therefore, the induction of each UPR component (e.g., BiP) is likely to be dependent on its intrinsic set-point for biological activation, specific cell context, and the defined ability of an agent to induce ER stress over time.

### Effect of CS exposure on the IRE1 branch of UPR in normal human lung cells

**Figure 8:**
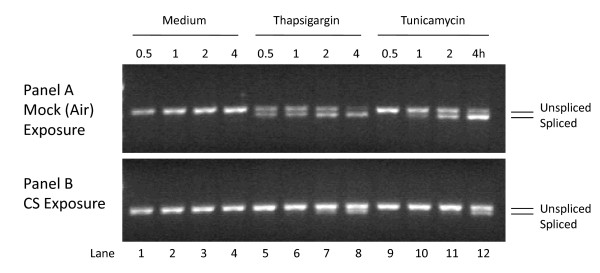
**Cigarette smoke inhibits XBP1 splicing**. A549 cells were exposed to air (**Panel A**, mock treatment) or 2R4F cigarette smoke (**Panel B**) for 20 minutes with 35 cc puffs diluted in 250 cc air. Cells were then placed in fresh media with or without 1 uM thapsigargin or 10 μg/ml tunicamycin for the time periods specified, followed by cell lysis and RNA purification. Spliced and unspliced XBP1 mRNA was detected using PCR methodology. Spliced XBP1 is 398 base pairs and the unspliced variant is 434 base pairs. For each lane the extent of splicing was quantified as described in the Methods section and is presented in additional file 3 – **Supplemental Table S3: Suppression of XBP1 splicing by cigarette smoke**.

The third UPR activation pathway is mediated by a cascade of events triggered by the dimerization and autophosphorylation of the IRE1 transmembrane protein. Under non-stressful conditions, IRE1 is inactive, however upon ER stress the conformational alteration of IRE1 via phosphorylation exposes a ribonuclease capability that removes an intron from XBP1 mRNA (denoted XBP1^S^), resulting in the generation of a functional protein that is a potent transcriptional regulator of genes involved in protein folding and degradation, two necessary mechanisms needed to restore ER homeostasis. The impact of CS exposure on XBP1 splicing was assessed. First, we determined the ability of A549 cells to undergo typical XBP1 splicing upon ER stress. Figure 8 (Panel A, lanes 1–4) shows that under non-stressed conditions (i.e., air exposure) only the unspliced form of XBP1 mRNA is detectable during the time-frame examined (30 min – 4 h) as expected. Figure 8 (Panel A, lanes 5–12) show that when A549 cells are exposed either to the ER stressors thapsigargin (which inhibits the ER Ca^2+ ^pump) or tunicamycin (which blocks N-linked glycosylation), there is a significant increase in XBP1^S ^starting at 30 minutes and continuing for up to 4 hrs post-exposure with a concomitant decrease in the unspliced form. In contrast, however, upon exposure to 2R4F CS the XBP1 mRNA remains in its unspliced form (Panel B, lanes 1–4) during the 0 – 4 h post-exposure assessment period. Indeed, when CS-exposed samples collected up to 24 h post-exposure were analyzed, no splicing of XBP1 was observed (data not shown).  For the extent of splicing, see Additional File 3.

To determine if the lack of splicing is due to a minimal impact of CS on IRE1 activation or active interference with the splicing mechanism, we performed a dual-treatment experiment in which A549 cells were first treated with CS and subsequently with thapsigargin or tunicamycin. If the inhibition of XBP1 splicing by CS is a dominant effect then the observed ability of UPR-inducing agents to promote splicing should also be compromised. Figure 8 (Panel B) shows that when A549 cells were exposed to CS and subsequently treated with either thapsigargin or tunicamycin, the observed XBP1^S ^levels were significantly diminished, indicating that CS exerts a potent suppressive effect upon XBP1 mRNA splicing (e.g., compare Panel B, lanes 5–12 with Panel A, lanes 5–12). In order to confirm that this suppression was not restricted to malignant lung cells, NHBE cells similarly treated with either thapsigargin or tunicamycin and exposed to 2R4F CS displayed an almost identical pattern of XBP1 splicing suppression (data not shown). In order to confirm that this suppression was not restricted to 2R4F, we examined two popular commercial U.S. brands of 'full-flavor' and 'lights' cigarettes for suppression of XBP1 splicing and observed essentially identical results to that of 2R4F (data not shown). Thus, these data indicate that the ability of CS to suppress XBP1 splicing is not cell or cigarette type specific but more likely due to a general property of CS exposure. Since CS as well as thapsigargin and tunicamycin can induce eIF2α-phosphorylation to a similar degree and within a similar time frame (e.g., see Figures 1A and 1B above for CS and thapsigargin respectively; tunicamycin data not shown), the suppressive effect of CS on XBP1 splicing is not a global inhibitory effect on other UPR effector arms. Indeed, treatment with both CS and thapsigargin appears to induce higher levels of eIF2α phosphorylation than either treatment alone, i.e. the effects appear to be additive (data not shown).

Our microarray data provide additional results to support the observation that the CS-induced suppression of XBP1 splicing has biological relevance. Since XBP1 alone or in conjunction with ATF6 controls the expression of a number of UPR-relevant genes (e.g., EDEM, HRD1, HERPUD1, ERdj3, P58IPK, ERdj4, and RAMP4 [19]), we conjectured that the inhibition of XBP1 splicing by CS would also prevent these transcripts from being expressed or upregulated. Of these seven genes, five of them (i.e., EDEM, HRD1, ERdj3, P58IPK, and RAMP4) showed no increase in expression above baseline (data not shown). The only XBP1 specific genes that showed an increase upon CS exposure were ERdj4 (DNAJB9) and HERPUD1, which increased 4-fold and 2.5-fold, respectively, over their expression levels in mock-treated cells at four hours post-exposure. Whether this indicates that there are additional mechanisms that complement XBP1 activation that can result in the expression of ERdj4 and HERPUD1 (as has been reported for HERPUD1 [66]), or if minimal amounts of spliced XBP1 are sufficient to induce their expression, remains to be determined. We note that Hengstermann and Muller [31] also found a lack of XBP1 splicing but concluded from their data that activation of IRE1 (which induces XBP1 splicing) by CS is negligible. However, our data suggest that the lack of XBP-1 splicing may in fact be due a direct inhibition by CS. Additional research should clarify the biological significance of the impact of CS on the IRE1-XBP1 pathway.

### Contribution of vapor and particulate phase constituents to eIF2α phosphorylation

**Figure 9:**
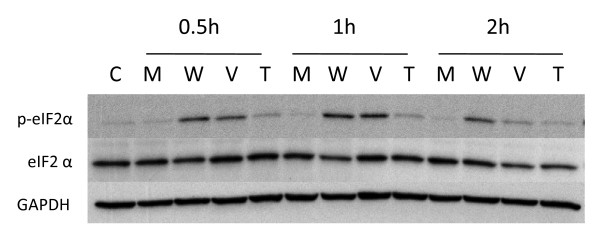
**Relative effect of vapor and particulate ('tar') phases of cigarette smoke on eIF2α phosphorylation**. NHBE cells were exposed to air (mock treatment M), whole cigarette smoke (W), vapor phase (V) or particulate 'tar' phase (T) from 2R4F cigarettes, after which the cells were placed in fresh medium and returned to the incubator for the time periods specified. Vapor phase was created by passing whole smoke through a Cambridge pad filter to trap the particulate matter. Particulate 'tar' phase was achieved by passing the whole smoke through 450 mg activated carbon to remove vapor phase components. Duration of whole smoke and vapor phase exposures was 20 minutes. Duration of particulate 'tar' phase exposures was 25 minutes in order to deliver an amount of particulate matter equivalent to that of the whole smoke exposure as some particulate matter is lost upon transit through the activated carbon. In all exposures the 35 cc puffs were diluted in 250 cc air. Western blots of whole cell lysates were probed with antibodies to phosphorylated eIF2α, eIF2α, and GAPDH.

CS is composed of a vapor phase and a particulate phase. The vapor phase is arbitrarily defined as that portion of smoke that passes through a Cambridge glass fiber filter, while the particulate phase is that portion that is retained by the filter [67]. The vapor phase is primarily a mixture of gases (i.e., nitrogen, oxygen, and carbon dioxide) as well as volatile and semi-volatile compounds such as carbon monoxide, acetone, nitrogen oxides, while the particulate phase contains a wide variety of condensed organic compounds (i.e., 'tar') that include nicotine and a large number of toxins, carcinogens, cocarcinogens, mutagens, and reactive organic and inorganic molecules [67]. This particulate phase contains the majority of tobacco smoke compounds [at least 80] for which there is sufficient evidence of carcinogenic potential in humans [68-70]. Presumably, the inherent chemical complexity of cigarette smoke results in an equally complex biological response involving a number of signaling pathways and checkpoints that respond to cellular and genomic stress in exposed tissues [71]. Few studies have attempted to address the synergistic relationships between the thousands of individual compounds that constitute the various classes of carcinogens in the vapor and particulate phases of tobacco smoke on gene expression. Thus, we assessed whether the fractionated vapor and particulate phases of CS from 2R4F cigarettes were also capable of inducing eIF2α phosphorylation as was whole CS. Figure 9 shows that the vapor phase was as efficient at inducing eIF2α phosphorylation in NHBE cells as was whole smoke. In contrast, the particulate phase appeared to be much less efficient at inducing eIF2α phosphorylation than either the vapor phase or whole CS. As previously observed for whole CS, there was no alteration in the total level of eIF2α after exposure to either the vapor or particulate phases of CS.

### Antioxidants prevent activation of UPR by CS

**Figure 10:**
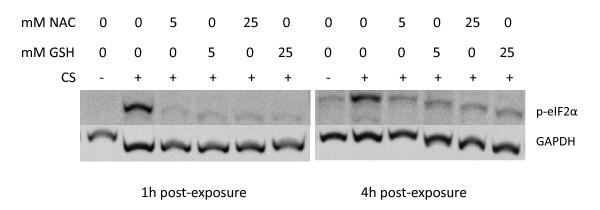
**Effect of antioxidant treatment on eIF2α phosphorylation following cigarette smoke exposure**. A549 cells were exposed to air (mock treatment) or 2R4F cigarette smoke for 20 minutes with 35 cc puffs diluted in 250 cc air, with or without concurrent treatment with NAC or GSH (at 5 and 25 mM concentrations), after which the cells were placed in fresh medium and returned to the incubator for the time periods specified. Western blots of whole cell lysates were probed with antibodies to phosphorylated eIF2α and GAPDH.

The results shown in Figure 9 indicated that the vapor phase is as efficient as whole CS in inducing ER stress and the phosphorylation of eIF2α. Since a primary constituent of the vapor phase of CS is a large amount of highly reactive organic and inorganic substances that either are free radicals or reactive species (such as ^•^O_2_^-^, ^•^NO, ^•^OH, etc), and more stable organic reactive species/oxidants (such as phenols, hydroquinone, epoxides, etc.) that can cause a marked imbalance in an individual's redox state and an overall increase in oxidative stress in the respiratory tract [72-75], we were interested in determining if free radical scavengers could effectively suppress the induction of eIF2α phosphorylation and the activation of the UPR program. Figure 10 shows that the thiol N-acetyl-L-cysteine (NAC), a potent free radical scavenging compound with antioxidant activity, can completely prevent CS-induced phosphorylation of eIF2α at 1 h or 4 h post-exposure in A549 cells. NAC is freely taken up by cells, and can impact a broad range of compounds, many of which cause some form of oxidative stress. For example, NAC can scavenge several reactive oxygen species (ROS) generated in CS such as ^•^O_2_^-^, ^•^OH, and H_2_O_2_, as well as directly bind and attenuate various reactive CS compounds that can generate free radicals intracellularly (e.g. aldehydes, epoxides, quinones, etc.). Figure 10 also shows that treatment with reduced glutathione (GSH), a free radical scavenger that does not cross the cell membrane, also suppresses the ability of CS to induce eIF2α phosphorylation.

### Contribution of vapor and particulate phase constituents to inhibition of XBP1 splicing

**Figure 11:**
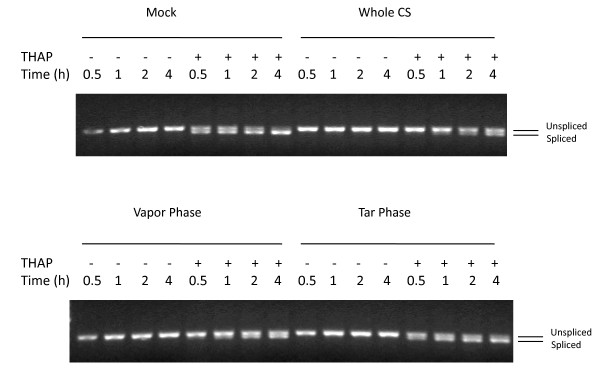
**Contribution of vapor and particulate phases of CS to XBP1 splicing inhibition in A549 cells**. A549 cells were exposed to air (mock treatment), whole cigarette smoke (CS), vapor phase, or particulate 'tar' phase from 2R4F cigarettes as described in the legend for Figure 6, then placed in fresh media with or without 1 uM thapsigargin (THAP) and incubated for the time periods specified. PCR was used to determine the relative amounts of spliced and unspliced XBP1. For each lane the extent of splicing was quantified as described in the Methods section and is presented in additional file 3 – **Supplemental Table S3: Suppression of XBP1 splicing by cigarette smoke**.

Figure 11 shows A549 cells exposed to particulate phase, vapor phase, and whole smoke from the reference cigarette 2R4F and treated with thapsigargin to assess inhibition of splicing. Similar to that observed with eIF2α phosphorylation, the vapor phase was as efficient at suppressing XBP1 splicing as whole smoke. In contrast, the particulate phase of cigarettes appeared not to inhibit splicing, suggesting that the inhibitory effect of CS is due to one or more vapor phase components.

### Expression of phospho-eIF2α, eIF2α, and BiP in human lung cancers

The data presented above indicate that in vitro exposure of lung cells to CS induces the phosphorylation of eIF2α but does not increase the expression of either total eIF2α or the major ER-stress induced chaperone protein BiP up to 24 hrs post-exposure. In order to determine the expression levels of these proteins in vivo, we next examined archival specimens of lung cancers. Expression of phospho-eIF2α, eIF2α, and BiP was assessed by immunohistochemistry on tissue arrays containing 360 cores representing 120 different histopathological classes of human lung neoplasms and normal tissue controls (see Table 1). We assessed the data in several ways. First, we determined if there were differences in the expression of phospho-eIF2α, eIF2α, and BiP between specimens of malignant and non-malignant lung tissue specimens. Second, we determined if there were differences in the expression of these three proteins between the malignant and non-malignant cellular compartments of each individual lung cancer specimen. Third, we determined if there was any meaningful correlation between expression levels of these three proteins and any clinicopathologic, demographic or smoking history parameter. For all three proteins (i.e., phospho-eIF2α, eIF2α, and BiP) we found that positive staining was strictly limited to the cytoplasm of all tissues examined. In addition, we did not observe any nuclear staining in any normal or malignant tissue, or any staining with an irrelevant control antibody.

### phospho-eIF2α

**Figure 12:**
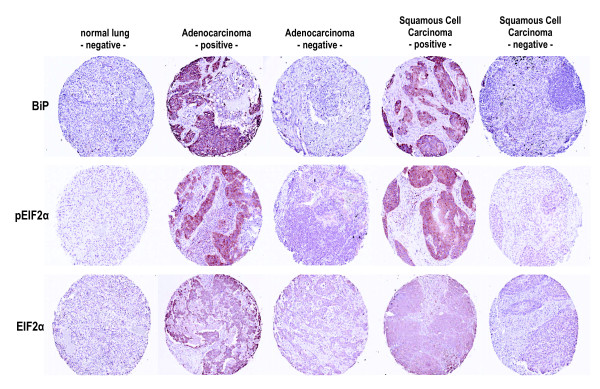
**Representative immunohistochemical expression features of phospho-eIF2α, eIF2α, and BiP proteins in different human lung lesions**. The proteins assessed by immunohistochemistry are designated in rows, while the columns depict expression in normal lung tissues, representative positive (ISI values of > 6–9) and negative NSCLCs, and representative positive (ISI values of > 6–9) and negative Small Cell Carcinomas. Magnification, ×200.

Representative immunohistochemical features of phospho-eIF2α staining are shown in Figure 12. Table 2 summarizes the immunoreactivity data and the statistical analysis detailed in Table 3 indicates a significant difference (*p *= 0.0025) between the mean phospho-eIF2α immunohistochemical staining indices (ISIs) for the non-small cell carcinoma diagnostic group and the normal diagnostic group. Of the 93 assessable cases of NSCLCs, 52 (55.9%) showed a significant increase in expression of phospho-eIF2α with an ISI between > 1–12 with many of these showing intense staining. There were no statistically significant differences in phospho-eIF2α expression between the normal diagnostic group and either the small cell lung carcinoma (SCLC) or mixed carcinoma (MC) diagnostic groups. Table 4 compares the ISI levels in the tumor compartment to the ISI levels in the normal compartment for each of the carcinoma diagnostic groups. The data indicate a statistically significant difference in expression of phospho-eIF2α between the malignant and non-malignant cellular compartments of both the NSCLC (*p *= < 0.0001) and MC (*p *= 0.0324) diagnostic groups, but not the SCLC (*p *= 0.1833) diagnostic group.

**Table 2:** Expression of phospho-eIF2α in normal and malignant lung specimens.

			Immunohistochemical Staining Index (ISI)				
			
Histology	Cellular Compartment	# of Cases	0–1^1^	> 1–3	> 3–6	> 6–9	> 9–12
Normal Lung Tissue	Normal cell compartment	8	8	0	0	0	0
Non-Small Cell Carcinoma^2^		93					
	Tumor cell compartment		41	18	22	9	3
	Normal cell compartment		87	4	2	0	0
Small Cell Carcinoma^2^		4					
	Tumor cell compartment		2	0	1	1	0
	Normal cell compartment		4	0	0	0	0
Mixed Carcinoma Types^2^		8					
	Tumor cell compartment		5	0	2	1	0
	Normal cell compartment		8	0	0	0	0

^1^An ISI value of 0–1 is considered negative, while an ISI value of > 1 is considered positive, with the 4 groups (i.e., > 1–3, > 3–6, > 6–9, > 9–12) indicate an increasing level of expression intensity and rate.

^2^For each tissue specimen tumor cells and non-tumor cells were scored separately for percent immunohistochemical reactivity and staining intensity as described in the Methods Section. The product of the percentage and intensity scores was used to generate an immunohistochemical staining index (ISI) for each cellular component (i.e., tumor and non-tumor) of each specimen.

**Table 3:** phospho-eIF2α protein expression.

Diagnostic Group	N	Mean +/- SD	P-value
Normal	8	0	--
Small Cell Carcinoma	4	3.00 ± 4.13	0.0875
Non-Small Cell Carcinoma	93	2.61 ± 2.77	0.0025*
Mixed Carcinoma	8	1.79 ± 2.39	0.0879

Mean immunostaining levels (± SD) and results of statistical testing for differences between the normal diagnostic group and each carcinoma type diagnostic group.

*Statistically significant (α = 0.05)

**Table 4:** phospho-eIF2α protein expression.

		Cellular Compartment		
			
Diagnostic Group	N	Normal	Tumor	P-value
Small Cell Carcinoma	4	0.21 ± 0.42	3.00 ± 4.13	0.1833
Non-Small Cell Carcinoma	90	0.24 ± 0.72	2.61 ± 2.77	< 0.0001*
Mixed Carcinoma	8	0.04 ± 0.12	1.79 ± 2.40	0.0324*

Mean immunostaining levels (± SD) and results of statistical testing for differences between the normal and tumor cell compartments for each diagnostic group.

*Statistically significant (α = 0.05)

### eIF2α

Representative immunohistochemical features of eIF2α staining are shown in Figure 12. Table 5 summarizes the immunoreactivity data and the statistical analysis shown in Table 6 indicates a significant difference between the mean eIF2α ISIs for the NSCLC diagnostic group and the normal diagnostic group (*p *= 0.0002), and between the MC diagnostic group and the normal diagnostic group (*p *= 0.0017). There was no statistically significant difference between the mean eIF2α ISI for SCLC diagnostic group and the normal diagnostic group (*p *= 0.5571). Of the 94 assessable cases of NSCLCs, 57 (60.6%) showed a considerable increase in expression of eIF2α with an ISI between > 1–12, although the intensity of protein expression was considerably less than that seen for phospho-eIF2α. Seven of eight assessable cases of MCs (87.5%) showed a statistically significant increase in expression of eIF2α with an ISI between > 1–12. Table 7 compares the ISI levels for eIF2α in the tumor cell compartment to the ISI levels in the normal cell compartment for each of the carcinoma diagnostic groups. The data indicate a statistically significant difference in expression of eIF2α between the malignant and non-malignant cellular compartments in both the NSCLC (*p *= < 0001) and MC (*p *= 0.0217) diagnostic groups but not for the SCLC (*p *= 0.923) diagnostic group.

**Table 5:** Expression of eIF2α in normal and malignant lung specimens.

			Immunohistochemical Staining Index (ISI)				
			
Histology	Cellular Compartment	# of Cases	0–1^1^	> 1–3	> 3–6	> 6–9	> 9–12
Normal Lung Tissue	Normal cell compartment	8	2	6	0	0	0
Non-Small Cell Carcinoma^2^		94					
	Tumor cell compartment		37	41	14	1	1
	Normal cell compartment		65	27	2	0	0
Small Cell Carcinoma^2^		5					
	Tumor cell compartment		4	1	0	0	0
	Normal cell compartment		5	0	0	0	0
Mixed Carcinoma Types^2^		8					
	Tumor cell compartment		1	6	1	0	0
	Normal cell compartment		7	1	0	0	0

^1^An ISI value of 0–1 is considered negative, while an ISI value of > 1 is considered positive, with the 4 groups (i.e., > 1–3, > 3–6, > 6–9, > 9–12) indicate an increasing level of expression intensity and rate.

^2^For each tissue specimen tumor cells and non-tumor cells were scored separately for percent immunohistochemical reactivity and staining intensity as described in the Methods Section. The product of the percentage and intensity scores was used to generate an immunohistochemical staining index (ISI) for each cellular component (i.e., tumor and non-tumor) of each specimen.

**Table 6:** eIF2α protein expression.

Diagnostic Group	N	Mean +/- SD	P-value
Normal	8	0	--
Small Cell Carcinoma	5	0. 39 ± 0.67	0.5571
Non-Small Cell Carcinoma	94	1.62 ± 1.34	0.0002*
Mixed Carcinoma	8	1.73 ± 1.24	0.0017*

Mean immunostaining levels (± SD) and results of statistical testing for differences between the normal diagnostic group and each carcinoma type diagnostic group.

*Statistically significant (α = 0.05)

**Table 7:** eIF2α protein expression.

		Cellular Compartment		
			
Diagnostic Group	N	Normal	Tumor	P-value
Small Cell Carcinoma	5	0.28 ± 0.18	0.39 ± 0.67	0.923
Non-Small Cell Carcinoma	93	0.90 ± 0.72	1.62 ± 1.34	< 0.0001*
Mixed Carcinoma	8	0.81 ± 0.27	1.73 ± 1.24	0.0217*

Mean immunostaining levels (± SD) and results of statistical testing for differences between the normal and tumor cell compartments for each diagnostic group.

*Statistically significant (α = 0.05)

### BiP

Representative immunohistochemical features of BiP staining are shown in Figure 12. Table 8 summarizes the immunoreactivity data and the statistical analysis shown in Table 9 indicates a significant difference between the mean BiP ISIs for the NSCLC diagnostic group and to the normal diagnostic group (*p *= < 0.0001), and for the MC diagnostic group and the normal diagnostic group (*p *= < 0.0001). There was no statistically significant difference between the mean BiP ISIs for SCLC diagnostic group and the normal diagnostic group (*p *= 0.3299). Of the 95 assessable cases of NSCLCs, 83 (87.3%) showed a significant increase in expression of BiP with an ISI between > 1–12. Of the 8 assessable cases of MCs all 8 (100%) showed a significant increase in expression of BiP with an ISI between > 1–12 and, as observed for phospho-eIF2α, many of these lung cancer cases displayed intense staining patterns. Table 10 compares the ISI levels in the tumor cell compartment to the ISI levels in the normal cell compartment for each carcinoma diagnostic group. The data indicate a statistically significant difference in expression of phospho-eIF2α between the malignant and non-malignant cellular compartments in both the NSCLC (*p *= < 0.0001) and MC (*p *= < 0.0001) diagnostic groups but not in the SCLC (*p *= 0.6116) diagnostic group.

**Table 8:** Expression of BiP in normal and malignant lung specimens.

			Immunohistochemical Staining Index (ISI)				
			
Histology	Cellular Compartment	# of Cases	0–1^1^	> 1–3	> 3–6	> 6–9	> 9–12
Normal Lung Tissue	Normal cell compartment	8	6	2	0	0	0
Non-Small Cell Carcinoma^2^		95					
	Tumor cell compartment		12	30	26	19	8
	Normal cell compartment		58	27	7	3	0
Small Cell Carcinoma^2^		5					
	Tumor cell compartment		4	0	0	1	0
	Normal cell compartment		3	2	0	0	0
Mixed Carcinoma Types^2^		8					
	Tumor cell compartment		0	3	2	2	1
	Normal cell compartment		7	1	0	0	0

^1^An ISI value of 0–1 is considered negative, while an ISI value of > 1 is considered positive, with the 4 groups (i.e., > 1–3, > 3–6, > 6–9, > 9–12) indicate an increasing level of expression intensity and rate.

^2^For each tissue specimen tumor cells and non-tumor cells were scored separately for percent immunohistochemical reactivity and staining intensity as described in the Methods Section. The product of the percentage and intensity scores was used to generate an immunohistochemical staining index (ISI) for each cellular component (i.e., tumor and non-tumor) of each specimen.

**Table 9:** BiP protein expression.

Diagnostic Group	N	Mean ± SD	P-value
Normal	8	0	--
Small Cell Carcinoma	5	1.37 ± 3.06	0.3299
Non-Small Cell Carcinoma	95	4.39 ± 2.92	< 0.0001*
Mixed Carcinoma	8	5.27 ± 3.20	< 0.0001*

Mean immunostaining levels (± SD) and results of statistical testing for differences between the normal diagnostic group and each carcinoma type diagnostic group.

*Statistically significant (α = 0.05)

**Table 10:** BiP protein expression.

		Cellular Compartment		
			
Diagnostic Group	N	Normal	Tumor	P-Value
Small Cell Carcinoma	5	0.90 ± 0.50	1.37 ± 3.06	0.6116
Non-Small Cell Carcinoma	95	1.26 ± 1.48	4.39 ± 2.92	< 0.0001*
Mixed Carcinoma	9	0.37 ± 0.51	5.27 ± 3.20	< 0.0001*

Mean immunostaining levels (± SD) and results of statistical testing for differences between the normal and tumor cell compartments for each diagnostic group.

*Statistically significant (α = 0.05)

### Correlation with Clinicopathological Variables

Expression levels of eIF2α, phospho-eIF2α, and BiP in tumor cells were assessed for any significant association with a clinicopathologic, demographic, or smoking history parameter (i.e., age, gender, race, smoking history, number of cigarettes/day, AJCC/UICC stage, and pT/pN category). The only correlation found in these data was a statistically significant increase in BiP in the NSCLC diagnostic group with increasing age (*p *= 0.0187). Finally, we assessed whether there was any concordance in expression of eIF2α, phospho-eIF2α, and BiP in any tumor cell compartment. A Spearman rank correlation analysis [76] indicated three statistically significant correlations for the increased expression of: 1) phospho-eIF2α and eIF2α (*p *= 0.0071) in patients with NSCLCs; 2) eIF2α and BiP (*p *= < 0.0001) in patients with NSCLCs; and 3) phospho-eIF2α and eIF2α (*p *= 0.0360) in patients of the MC diagnostic group.

## Discussion

The pyrolysis process in cigarettes generates a highly complex mixture of reactive gases and suspended particulate matter containing a wide range of carcinogens, tumor promoters, toxins, and free radicals which cause direct and indirect damage to the genome [77,78], transcriptome [79,80], and proteome [81] of cells in the aerodigestive tract of smokers on a daily basis [82,83]. This incessant cycle of tissue injury and repair is presumed to be a major contributor to the development of lung cancer [75,84-86]. However, a precise biological understanding of the nature and temporal sequence of specific damaging events that drive the formation and progression of this disease remains elusive [87-91]. Compelling data support the conclusion that one prominent form of CS-induced airway damage and a key etiological factor in lung cancer is chronic oxidative stress and resulting inflammation [72-75,84-86,92,93]. For example, 1) lung cancer is increased in patients with chronic inflammation [84,94-96]; 2) polymorphisms and mutations in genes regulating the inflammatory process are linked to lung cancer risk [97,98]; 3) gene and proteomic studies of smoke-exposed airway epithelium consistently shows the upregulation of genes that respond to oxidants [30,31,99]; 4) the cyclooxygenase-2 (COX-2) isoenzyme, a key player in diverse pro-inflammatory conditions, is frequently up-regulated in lung neoplasms and correlates with a poor prognosis [100,101]; 5) non-steroidal anti-inflammatory drugs (e.g., selective COX-2 inhibitors such as Celecoxib) can reduce the relative risk of lung cancer [102-105]; 6) CS can depress levels of endogenous antioxidants such as glutathione [74,106-108]; 7) synergy between pro-oxidant beta-carotene cleavage products and resulting oxidative stress is believed responsible for the increased rates of lung cancer in long-term smokers enrolled in both the ATBC (Alpha-Tocopherol Beta-Carotene) and CARET (beta-Carotene and Retinol Efficacy) chemoprevention trials [109]; 8) a mouse model system suggests that oxidative stress induced in the lung by CS vapor phase components is a key player in lung oncogenesis [85,110]; and 9) we have previously shown that an early genomic defect caused by the copious amounts of free radical generators in CS is the induction of DNA double-strand DNA breaks (DSBs), which are probable tumorigenic lesions in multiple cancers including those of the lung [111-113], and that free radical scavenging antioxidants can prevent these DSBs [3,5].

Although a detailed understanding of the molecular mechanisms that directly link oxidative stress, inflammation, and CS-induced pathologies is still lacking, a wealth of data suggest that the large amounts of free radicals and reactive species (such as ^•^O_2_^-^, ^•^NO, ^•^OH, etc) in the gas phase, and more stable organic reactive species/oxidants (such as phenols, hydroquinone, epoxides, etc.) in the particulate phase of CS, overwhelm the respiratory tract's steady-state antioxidant capacity causing a marked imbalance in its redox status [114-116]. This persistent exogenous source of oxidative stress is amplified and augmented by an endogenous chronic host inflammatory response at the sites of tissue damage that provides additional quantities of reactive oxygen and nitrogen compounds (e.g., H_2_O_2 _and ^•^NO), and reactive intermediates such as peroxynitrite (ONOO^- ^[86,117,118]. Combined, these sources of reactive species can cause significant cellular and tissue damage that the cell responds to in several ways. One mechanism is to alter the expression of genes that attenuate the effects of oxidative stress. For example, as shown in additional file 1 – **Supplemental Table S1: Cigarette smoke signature genes**, CS exposure induces the expression of: a) thioredoxin reductase 1, a component of a ubiquitous thiol oxidoreductase system that protects the cell from oxidative stress; b) heme oxygenase I (HO-1), an enzyme that catabolizes heme containing proteins with the subsequent production of free iron, CO, and bilirubin; c) ferritin, which sequesters reactive iron molecules and d) NAD(P)H:quinone acceptor oxidoreductase 1 (NQO1), a cytosolic flavoenzyme that catalyzes reduction of quinones to hydroquinones and is part of the oxidative stress response [119]. Each of these CS-responsive genes is frequently upregulated in a range of cancers including lung carcinomas [120-123]. Our data further show that CS causes an increase in the expression of genes pivotal to redox homeostasis as well as the synthesis of glutathione: gamma glutamylcysteine ligase, catalytic and modifier subunits. Chronic depletion in glutathione related antioxidant enzymes by CS can lead to lung damage and disease [124,125]. Since proteins are major targets of oxidative damage, another predictable biological result would be an accumulation of misfolded, aggregated, or cross-linked proteins in the ER of the respiratory tract which responds by activating a wide array of stress responsive genes in order to restore homeostasis [126]. Support for this hypothesis comes, in part, from the data presented in this study showing that CS causes ER stress and activates the UPR pathway via phosphorylation of eIF2α in a PERK-dependent manner in human lung cells, which supports a similar conclusion reached in two recent papers [30,31]

Our data further show that reactive radical species in both whole smoke and the vapor phase are a dominant CS-component causing ER stress [127]. A recent publication by Hengstermann and Muller has concluded that the CS gas phase component acrolein is a major inducer of ER stress [31]. The ability of both GSH (the major intracellular antioxidant) and NAC, a synthetic acetylated form of the amino acid L-cysteine and a thiol-containing antioxidant, to scavenge CS-generated reactive oxygen species (ROS) (e.g., ^•^O_2_^-^, H_2_O_2_, and ^•^OH) [128,129] or sequester reactive CS compounds that spawn free radicals intracellularly (*e.g*., aldehydes, epoxides, quinones, etc.) further supports the mechanistic relationship between CS-induced reactive species and ER stress [130]. In these in vitro experiments it is perhaps not surprising that the particulate phase of CS did not induce the UPR. This is presumably because many of the compounds that have reactive potential in the particulate phase require either enzymatic activation or generation via quinone/hydroquinone redox coupling in the presence of oxygen over time [127,131]. Thus, the particulate phase does not result in a bolus of free radicals at short time periods but rather chronic low-level radical generation over extended time periods [131,132]. Vapor phase components are inherently smaller/more reactive than particulate phase components and therefore have a higher likelihood of reacting with biological components immediately upon exposure as well as crossing phospholipid membranes more quickly than larger molecules. Consequently, it is probable that since we exposed cells to CS for only 15–20 minutes, there is insufficient time for reactive species to form within the cell and induce the UPR pathway. Presumably, this is not the case in vivo where chronic cigarette smoking would provide sufficient time for the reactive species in the particulate phase to impact protein structures and induce molecular circuits like the UPR.

Another interesting observation to emerge from this study is that CS significantly suppressed the splicing of the XBP1 mRNA to its active truncated form. The ability of CS to modulate the expression of XBP1, if mirrored in vivo in smokers, may have significant ramifications for the development and progression of lung neoplasms since XBP1 not only has a primary role in maintaining ER homeostasis [22,133], but also important regulatory functions in DNA damage and repair pathways, redox homeostasis and oxidative stress responses [22]. Thus, impairment of this key effector arm of the UPR pathway could have considerable detrimental short and long-term effects in smokers. The mechanism by which CS suppresses XBP1 splicing is not obvious. It is possible that one or more components of CS can attack the structure of the IRE1 transmembrane protein thereby disabling its ribonuclease function and subsequent splicing of the XBP1 mRNA to its transcriptionally active isoform. Another possibility is that CS activates a protein that inhibits elements of the ER-stress induced UPR pathway. For example, the Bax inhibitor-1 (BI-1) protein has been shown to suppress apoptosis [134], ER-stress related protein expression, and XBP1 splicing [135]. Moreover, it has been shown that, depending upon the subtype, some 43% to 82% of lung adenocarcinomas overexpress BI-1 [136]. Eliminating or down-regulating proapoptotic signals are a major step in the evolution of most, if not all, human malignancies. We are currently assessing if CS suppresses XBP1 splicing by inducing or augmenting BI-1 expression in lung cells. A recent paper has presented evidence showing that mouse 3T3 cells exposed to aqueous extracts of CS displayed only minute amounts of spliced XBP1 mRNA [31]. The conclusion of this paper was that CS did not activate the IRE1 sensor and therefore resulted in negligible XBP1 splicing. In contrast, we provide evidence that CS actively suppresses the splicing of XBP1. If this phenomenon occurs in vivo in smokers, it could have physiological relevance in terms of altering the balance between cell survival and cell death. Lin et al have recently shown that IRE1 and ATF6 activities were attenuated by persistent ER stress in human cells resulting in increased cell death [61]. Clearly, further research to determine if IRE1 activation and/or XBP1 splicing is corrupted in actual smokers would be valuable. Finally, we showed that the third effector arm of the UPR signaling pathway, ATF6, is also activated by CS as evidenced by cleavage of the ATF6 protein and the appearance of downstream transcriptional targets of ATF6 such as XBP1 and BiP.

If activation of the UPR program occurs in smokers on a daily basis due to oxidative stress, it could evolve into a long-term problem either because of persistent damage directly related to its activation, or because as a functional barrier to transformation and tumor progression, it provides an attractive target for disablement. Either scenario would allow the respiratory cell to sustain additional damage to mechanisms that maintain lung homeostasis which could eventually lead to neoplastic transformation [137,138]. A number of studies have suggested that habitual activation of UPR plays a major role in etiology of cancer as well as many other diseases [35,36,139,140]. Tumor cells are subjected to considerable internal stress due to genetic instability, hypoxia, signaling distortion, immune attack, and disorganized cross-talk with the surrounding normal microenvironment [141]. Many of these stressful situations can cause ER dysfunction from which the cell must defend itself. Consequently, a prevailing hypothesis is that upregulation of UPR components in malignant cells provides an anti-apoptotic, pro-survival advantage by increasing resistance to ER stress induced by endogenous sources (e.g., hypoxia, genetic instability, etc.) [10,36,64,142] or exogenous sources (such as chemotherapeutic drugs) [32,143]. Evidence to support this idea comes from numerous studies showing the increased expression of one or more UPR-relevant proteins in multiple tumors including those of the breast (i.e., BiP [144]), liver (ATF6, XBP1 and BiP [145]), stomach (BiP [146]), brain (BiP [143]), esophagus (Grp94 [147]), and lung (BiP and Grp94 [148,149]). Our analysis of lung cancer tissue specimens is also consistent with this hypothesis. For example, we found that compared to non-malignant lung tissue, there is a significant increase in expression of phospho-eIF2α in a majority of cases of NSCLCs (55.9%; *p *= 0.0025) but not in either SCLCs or MCs. We further found a significant increase in expression of BiP in a majority of cases of NSCLCs (87.5%; *p *= < 0.0001) and MCs (100%; *p *= < 0.0001), but not in SCLCs. The eIF2α protein was also overexpressed in a majority of cases of both NSCLCs (60.6%; *p *= 0.0002) and MCs (87.5%; *p *= 0.0017), but not in SCLCs. Rosenwald et al. have previously shown that eIF2α is frequently increased in bronchioloalveolar carcinomas (BAs) but only rarely in squamous cell carcinomas (SCCs) [150]. Since both BAs and SCCs are subgroups of NSCLCs that share the histological feature of being derived from lung epithelium [151], these data differ somewhat from ours which showed a statistically significant increase in eIF2α expression in the tumor cell compartments of SCCs as well as in the other major NSCLC histological subtypes (i.e., adenocarcinoma, adenosquamous carcinoma, and large cell carcinoma) for which we had a sufficient number of cases. Resolution of the differences between our results and that of Rosenwald et al. awaits further research. However, an important caveat to our data is that the small number of SCLC cases available for assessment (i.e., 5) could potentially obscure any reliable protein expression differences.

Although our data support the conclusion that modulators of the UPR pathway are chronically impacted in this tumor type, aside from a modestly significant increase in BiP in the NSCLC diagnostic group with increasing age (p = 0.0187), we found no obvious correlation with any histopathological criteria such as gender, pathologic stage, histological type, or TMN-status and the increased expression of eIF2α, phospho-eIF2α, and BiP. Uramoto et al. [149] similarly found no significant difference between BiP expression and any clinicopathological parameter. A recent lung cancer study by Wang et al. [148], though finding no correlation of increased expression of BiP with pathologic tumor type, did relate augmented expression in less differentiated tumors and in more advanced stage III tumors, both aspects predicting a poorer prognosis. However, Uramoto et al. [149] found increased expression of BiP in a majority of lung cancers but these patients had a better prognosis than those with BiP-negative cancers. In our current study, we could not determine the relationship between expression and prognosis due to a lack of detailed clinical outcome data on the patients from whom the tumor specimens we assessed originated.

Paradoxically, although our study showed that increased expression of eIF2α, phospho-eIF2α, and BiP are pathogenic features of lung cancers, none of these proteins identified lung cancers as having arisen in a smoker or nonsmoker. Does this mean that the induced expression of these UPR-related proteins is not directly related to CS exposure or, alternatively, that their induction is a general convergent feature of lung cancers regardless of the provoking stimulus? Since oxidative stress resulting from passive cigarette smoking is certainly one etiological factor in the development of lung cancers in nonsmokers [75,84,85,92,152,153] the latter interpretation is more likely. It is also possible that the UPR is induced in CS-exposed lung cells prior to malignancy but also subsequent to the development of a cancer that has regions of hypoxia. For example, it is well documented that hypoxia is present in a majority of human solid tumors, including lung cancers, and that hypoxic regions can have selective resistance to various therapeutic modalities [53,154]. Thus, one prosurvival mechanism of hypoxic tumor cells is to diminish protein translation and energy utilization, which can occur as a direct result of activation of the UPR pathway. Accordingly, induction of UPR via eIF2α phosphorylation is required for hypoxic cell survival and tumor growth [53]. In addition, hypoxia can also induce the expression of BiP [155]. Thus, it remains to be determined if the expression of peIF2α and BiP that we observed in a majority of human lung cancers occurs relatively late in their evolution as the result of hypoxic conditions, or reflects an early activated prosurvival mechanism in asymptomatic lung cells undergoing chronic ER stress due to CS or some other environmental contaminant. While our data to date do not strongly support either possibility, a recent study using a proteomic approach showed that lung samples from chronic smokers demonstrated a number of differentially expressed proteins compared to nonsmokers [30]. For example, several UPR proteins, including BiP and calreticulin, were found to be up-regulated in smokers. The conclusion of these data was that activation of UPR by CS may protect the lung from oxidant injury and the development of chronic obstructive pulmonary disease, a strong risk factor for the development of lung cancer.

In summary, while more studies are needed to clarify how chronic activation, expression, or dysregulation of key UPR-regulated proteins impact the trajectory of CS-induced lung disease, it seems highly likely that the UPR pathway is a one of several molecular mechanisms promoting tumor cell evolution that could be attenuated or reversed resulting in a more efficacious treatment strategy for lung cancers [87-90,156-158]. Targeting one or more UPR effectors, either unilaterally or in combination with conventional cytotoxic drugs, may be a particularly important treatment opportunity since the current standard of care for patients with advanced lung cancer remains disappointing [33,35,36,137,156]. Direct support for this proposition has recently come from a study showing that bortezomib (PS-341, Velcade™), a potent proteasome inhibitor currently approved for the treatment of multiple myeloma, sensitizes pancreatic cancer cells to ER stress-induced apoptosis and strongly enhances the anticancer activity of cisplatin [159].

## Conclusion

The following conclusions can be drawn from these data. First, since different types of cigarettes are equally effective at inducing ER stress and activating the UPR pathway, it suggests that the toxicity and chemical composition of CS is relatively constant across different brands of cigarettes, an observation consistent with epidemiological and exposure studies that find no essential difference in lung cancer risk among long-term smokers of different cigarette types (i.e., ultralight, light, or full-flavor)[44,46], and no significant quantitative differences in markers of carcinogen and nicotine uptake among these smokers [46,160]. Second, the induction of ER stress in combination with chronic activation of multiple effectors of the UPR pathway by CS may play a pivotal role in the etiology and/or progression of lung cancers and other pulmonary diseases. And third, perturbations in phospho-eIF2α, as well as BiP and eIF2α, may have diagnostic and/or therapeutic potential in the clinical management of patients with various lung malignancies.

## Abbreviations

AJCC: American Joint Committee on Cancer; ATF6: activation transcription factor 6; BiP: binding immunoglobulin protein; CS: cigarette smoke; DTT: dithiothreitol; ER: endoplasmic reticulum; eIF2α: eukaryotic translation initiation factor 2-alpha; phospho-eIF2α: phospho-eukaryotic translation initiation factor 2-alpha; FTC: federal trade commission; ISI: immunohistochemical staining index; NSCLC: non-small cell lung carcinoma; PCR: polymerase chain reaction; SCC: squamous cell carcinoma; UPR: unfolded protein response; XBP1: X-box binding protein.

## Competing interests

Vector Research LLC is an affiliate of Vector Tobacco Inc which funded this research.

## Authors' contributions

EJ performed the microarray analysis, participated in the design of the study, and the writing of the manuscript; AS, LS, JY, and DG contributed to the data generation and participated in study design; and APA participated in the design, coordination, and performance of the study, assisted in the writing of the manuscript, and funded the study. All authors have read and approved the final manuscript.

## Pre-publication history

The pre-publication history for this paper can be accessed here:



## Supplementary Material

Additional file 1 **Supplemental Table S1: Cigarette smoke signature genes**. NHBE cells were treated with whole smoke from a reference cigarette and two of the largest-selling commercial brands in the United States. Cigarette smoke "signature genes" (the list of genes showing significant change in expression at 2 h, 4 h, and 24 h post-exposure for all three cigarette treatments) are shown for each time point. Click here for file

Additional file 2 **Supplemental Table S2: Functional annotation clustering of cigarette smoke signature genes**. Cigarette smoke signature genes subjected to functional annotation clustering of Biological Process Gene Ontology categories using the algorithms on the NIH DAVID website. Only significant (mean p value < 0.01) clusters are listed. Click here for file

Additional file 3 **Supplemental Table S3: Suppression of XBP1 splicing by cigarette smoke**. Calculation of percent spliced XBP1 RNA. Gel photographs from PCR experiments presented in Figures 8 and 11 were analyzed as described in the Methods section and all gel bands quantified. After normalization, the percent splicing was calculated using the formula: spliced/(unspliced + spliced) × 100. Click here for file
